# Body Mass Index and Spinopelvic Alignment as Predictors of Incident Knee Osteoarthritis: An 8-Year Longitudinal Study from the TOEI Cohort of Older Japanese Women

**DOI:** 10.3390/jcm14207343

**Published:** 2025-10-17

**Authors:** Yuki Murakami, Mitsuru Hanada, Kazuki Nomoto, Kensuke Hotta, Yuki Yamagishi, Yukihiro Matsuyama

**Affiliations:** 1 Department of Orthopaedic Surgery, Hamamatsu University School of Medicine, Hamamatsu 431-3192, Japan; 2 Faculty of Informatics, Shizuoka Institute of Science and Technology, Shizuoka 420-0857, Japan

**Keywords:** knee osteoarthritis, risk factors, body mass index, spinal alignment, pelvic incidence–lumbar lordosis mismatch, longitudinal cohort

## Abstract

**Background/Objectives**: Knee osteoarthritis (KOA) is multifactorial, and longitudinal evidence isolating early predictors remains limited. We investigated predictors of incident KOA in community-dwelling older adult Japanese women. **Methods**: We analyzed 191 knees from 105 women aged ≥50 years (baseline Kellgren–Lawrence (KL) grade 0–1) and followed them for 8 years. Incident KOA was defined as KL ≥ 2 at the 8-year follow-up. Baseline measures included body mass index (BMI), physical function (one-leg stance, functional reach), Geriatric Locomotive Function Scale (GLFS-25), EuroQol 5-Dimension (EQ-5D) questionnaire, standing lateral whole-spine radiographs (sagittal spinopelvic parameters), and standing full-length anteroposterior (AP) lower-limb radiographs (coronal alignment parameters). Incident KOA was defined as KL ≥ 2 at follow-up. Group comparisons, multivariable logistic regression, and receiver operating characteristic analyses were conducted. **Results**: Incident KOA occurred in 58/191 knees (mean participant age 69.3 ± 6.1 years). Compared with non-incident knees, incident knees had higher BMI (23.8 vs. 21.1 kg/m^2^), higher GLFS-25, greater pelvic tilt and pelvic incidence minus lumbar lordosis (PI–LL) mismatch (11.5° vs. 5.3°), and lower EQ-5D, medial proximal tibial angle, and joint line obliquity. BMI was the strongest single predictor (area under the curve [AUC] 0.753). PI–LL mismatch showed limited standalone discrimination (AUC 0.596) but improved discrimination when combined with BMI (AUC 0.803). **Conclusions**: BMI was the primary predictor of incident KOA in this cohort. PI–LL mismatch, while not strongly discriminative alone, acted as a complementary marker consistent with sagittal-alignment-related mechanical stress. Results suggest that early screening and prevention should prioritize weight management, using spinopelvic parameters to refine risk stratification.

## 1. Introduction

Knee osteoarthritis (KOA) is a leading cause of pain, disability, and reduced quality of life (QOL) in older adults, particularly women [1,2,3]. With the increase in global aging, the burden of KOA is expected to rise dramatically, nearly doubling by 2050 [4,5]. Therefore, identifying modifiable risk factors—ideally prior to disease onset to enable primary prevention and risk-stratified screening—is essential for early intervention and disease prevention.

Previous epidemiological studies identified several risk factors for KOA, including older age, higher body mass index (BMI), female sex, history of knee injury, and impaired musculoskeletal function [6,7,8,9,10]. However, many reports have examined mixed outcomes, often conflating incident KOA with disease progression; focusing on incident KOA may help clarify the highly modifiable targets most relevant for prevention. Recently, attention has shifted toward biomechanical factors beyond the knee joint. Notably, spinal and pelvic alignment may influence knee joint loading [11,12,13]. For example, anterior trunk tilt and decreased lumbar lordosis may increase mechanical stress on the knees and contribute to the development of KOA [14,15,16]. In Asian populations, varus alignments of the proximal tibia may increase medial compartment loading and elevate the risk for KOA [17,18].

However, whether factors such as musculoskeletal decline and spinal alignment abnormalities contribute to incident KOA, or instead reflect secondary changes accompanying KOA progression, remains unclear. Additionally, these factors are frequently interrelated, complicating the isolation of their individual effects. Clarifying their predictive value requires longitudinal studies in individuals without advanced KOA. However, such cohort studies are limited. In particular, longitudinal studies controlling for sex and injury history are lacking.

To minimize the confounding effect of sex differences in joint morphology and KOA risk, this study focused exclusively on women. Therefore, this study aimed to identify the predictors of incident KOA through an 8-year longitudinal analysis of community-dwelling women aged ≥50 years in rural Japan.

## 2. Materials and Methods

This study was approved by our institutional ethics committee. Written informed consent was obtained from all participants, and the study adhered to the principles of the Declaration of Helsinki (1964).

This study was part of the TOEI study, a community health project conducted in Toei Town, Aichi, Japan. This ongoing biennial survey, initiated in 2012, targets healthy participants aged ≥50 years and includes physical function tests and radiographic evaluations. Participants were residents who were recruited through Toei Hospital or public bulletins. Reports from the TOEI cohort have been published previously [18,19,20].

Of the 225 individuals who participated in the 2012 and 2020 surveys, 148 women were included in the present analysis. The inclusion criteria were age ≥ 50 years and no history of spinal surgery. The exclusion criteria were incomplete questionnaire data (3 participants, 6 knees) and poor-quality radiographs or history of lower-limb surgery (8 participants, 15 knees). A total of 11 participants (21 knees) were excluded. Formal power analysis was not performed owing to the fixed cohort size.

The final analysis included 138 participants (275 knees), of which 191 knees from 105 participants were classified as Kellgren–Lawrence (KL) grade 0 or 1 in 2012 and included in the analysis. In cases where only one knee met this condition, the contralateral knee was classified as KL grade 2.

All participants provided their demographic data in 2012, including age, height, weight, and BMI, and underwent the following assessments. Data regarding occupational and sports history were not collected (a limitation of this study).

- Physical Function Evaluation

Physical function was evaluated using the one-leg standing time (with eyes open) and the Functional Reach Test (FRT). For the one-leg standing test, each leg was tested twice, and the longest standing time (up to 60 s) was recorded. For the FRT, participants extended one arm forward and leaned their upper body forward without moving their feet, and the maximum distance reached by the fingertips was measured in two trials. Although knee symptoms were not formally assessed, all participants completed the functional tests without difficulty.

- Questionnaires

Musculoskeletal function was evaluated using the self-administered 25-item Geriatric Locomotive Function Scale (GLFS-25) and the EuroQol 5-Dimension (EQ-5D) questionnaire. The GLFS-25 contains 25 items addressing mobility, activities of daily living, social function, and mental health domains, each scored 0–4 (total score 0–100, with higher scores indicating worse dysfunction) [21,22]. QOL was assessed using the EQ-5D, a standardized health status measure [23]. Higher GLFS-25 and lower EQ-5D index scores indicate greater musculoskeletal impairment.

- Radiographic Evaluation

Full-spine and full-length lower-limb radiographs were obtained. Standing lateral whole-spine radiographs were obtained with participants looking straight ahead and hands on the clavicles. Standing full-length anteroposterior (AP) lower-limb radiographs were acquired with the knees fully extended and the patellae facing forward. The X-ray tube was positioned 1.5 m from the participant. Images were analyzed using Surgimap Spine software (version 2.3.2.1; Nemaris, New York, NY, USA).

**Figure 1:**
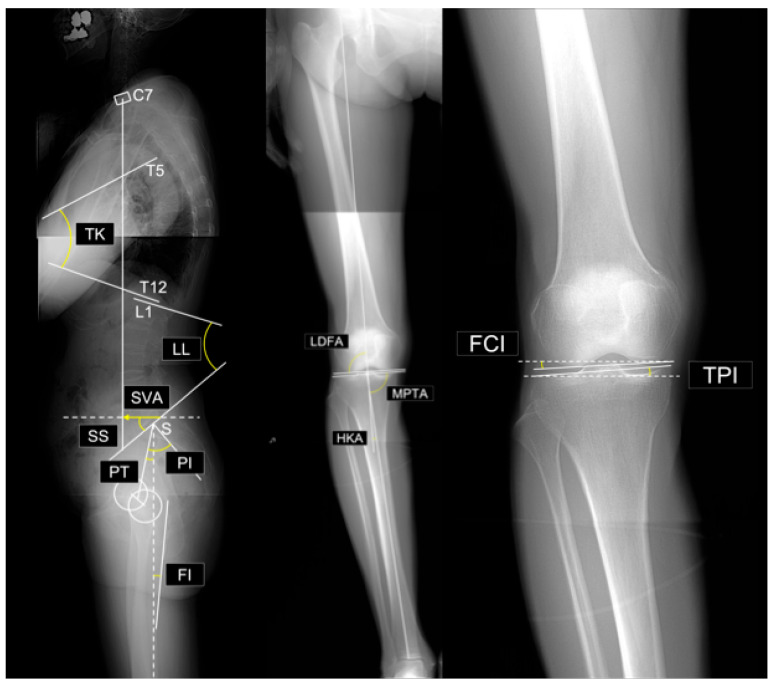
Evaluated radiographic parameters. Abbreviations: SVA, sagittal vertical axis; TK, thoracic kyphosis; LL, lumbar lordosis; SS, sacral slope; PT, pelvic tilt; PI, pelvic incidence; PI–LL, PI minus LL; FI, femoral inclination; LDFA, lateral distal femoral angle; MPTA, medial proximal tibial angle; HKA, hip–knee–ankle angle; FCI, femoral condyle inclination; TPI, tibial plateau inclination.

The radiographic parameters (see Figure 1) measured in this study were as follows:

Spinal and pelvic parameters:

- Sagittal vertical axis (SVA): The horizontal distance between the vertical plumb line dropped from the center of the C7 vertebral body and the posterosuperior corner of the sacrum.
- Thoracic kyphosis (TK): The angle between the superior endplate of T1 and the inferior endplate of T12.
- Lumbar lordosis (LL): The angle between the superior endplate of L1 and the superior endplate of S1.
- Sacral slope (SS): The angle between the superior endplate of S1 and a horizontal reference line.
- Pelvic tilt (PT): The angle between the line connecting the midpoint of the S1 superior endplate to the femoral head center and a vertical reference line
- Pelvic incidence (PI): The angle between a line perpendicular to the S1 superior endplate at its midpoint and the line connecting this midpoint to the femoral head center. By definition, PI = SS + PT.
- PI–LL mismatch: Difference between PI and LL (i.e., PI minus LL).
- Femoral inclination (FI): The angle between the femoral axis and the vertical reference line. Mean values from both sides were used.

Lower limb parameters:

- Lateral distal femoral angle (LDFA): The lateral angle between the femoral mechanical axis and distal joint line.
- Medial proximal tibial angle (MPTA): The medial angle between the tibial mechanical axis and proximal joint line.
- Arithmetic hip–knee–ankle angle (aHKA): Defined as MPTA minus LDFA, representing overall lower-limb alignment. Negative values indicate varus alignment.
- Joint line obliquity (JLO): Calculated as the sum of the MPTA and LDFA, reflecting the inclination of the joint line relative to the floor in a bipedal standing position. Values less than 180° indicate the apex distal configuration.
- Hip-knee-ankle angle (HKA): The angle between the mechanical axes of the femur and tibia. A value of 0° represents neutral alignment, and positive values indicate varus alignment.
- Joint line convergence angle (JLCA): The angle formed by the intersection of the distal femoral and proximal tibial joint lines. Positive values indicate a lateral opening.
- Femoral condyle inclination (FCI): The inclination of the femoral joint surface relative to the horizontal plane. Negative values indicate lateral distal inclination.
- Tibial plateau inclination (TPI): inclination of the tibial joint surface relative to the horizontal plane. Negative values indicate medial-distal inclination.

Radiographic analyses were conducted by two board-certified orthopedic surgeons. The intraobserver and interobserver reliability for all alignment parameters and KL grade was high with intraclass correlation coefficients (ICCs) >0.75 (range: 0.792–0.988).

- Group classification and analysis

Participants with KOA classified as KL grades 0 or 1 in 2012 were included in this study. Those with knees progressing to KL grade ≥2 by 2020 were classified as incident KOA (I group), while those remaining at KL grade 0 or 1 were classified as non-incident KOA (NI group). Mean values of each outcome measure were compared between groups I and NI. Multivariate analysis was performed to identify potential predictors and assess multicollinearity among the outcome measures. For the predictors that remained significant in the multivariate analysis, the optimal cut-off points were determined, and the corresponding cut-off values were calculated.

- Statistical Analysis

Continuous variables are expressed as mean ± standard deviation, and *t*-tests were used for group comparisons. To identify the predictive factors, multivariate analysis was performed using binary logistic regression. Stepwise selection was performed using the Wald test, with a significance level of *p* < 0.01. Receiver operating characteristic (ROC) curve analysis was used to assess diagnostic accuracy. Collinearity was assessed using variance inflation factors (VIFs), and Spearman’s rank correlation was used for correlation analysis. Scatter plots were generated for each variable identified as an independent predictor, with predicted probabilities from the logistic regression model overlaid. A *p*-value <0.05 was considered statistically significant. To enhance readability, in-text statistics are typically rounded to two decimals, whereas tables report values to four decimals. All performance indices and hypothesis tests were computed using full-precision estimates.

- Internal validation and performance

Harrell’s bootstrap optimism correction (1000 resamples) was used to estimate and remove overfitting bias (“optimism”) from apparent performance. We report the Brier score (mean squared error of predicted probabilities) and the Brier Skill Score (BSS = 1 − Brier_model/Brier_null), which reflects improvement over a null model that predicts the cohort incidence only. Calibration was summarized by the slope (ideal ≈ 1) and intercept (ideal ≈ 0) from regressing the outcome on the model’s logit predictions.

All computations for statistical testing, data processing, and figure generation were performed in MATLAB R2025a (MathWorks, Natick, MA, USA), employing built-in functions and add-on toolboxes.

## 3. Results

The mean age of the participants was 69.3 ± 6.1 years (52–82). Of 191 knees, 133 were non-incident (NI group) and 58 incident (I group). No cases of medial femoral condyle osteonecrosis or rheumatoid arthritis were observed in the I group. Incident knees showed higher weight and BMI (23.8 vs. 21.1 kg/m^2^), higher GLFS-25 scores and lower EQ-5D index values, greater PT and PI–LL mismatch (11.5° vs. 5.3°), and smaller MPTA and JLO (Table 1).

A binary logistic regression analysis was conducted using age, BMI, GLFS-25, EQ-5D, SVA, TK, SS, PT, PI–LL mismatch, MPTA, and LDFA as explanatory variables. BMI and PI–LL mismatch were selected as significant predictors of incident KOA, and the following base model was constructed:

logit = −9.0544 + 0.35634 × BMI + 0.029861 × PI–LL mismatch

With an interaction term, the extended model was

logit = −11.445 + 0.45543 × BMI + 0.28648 × PI–LL mismatch − 0.011004 × BMI × PI–LL mismatch

(Table 2).

**Figure 2:**
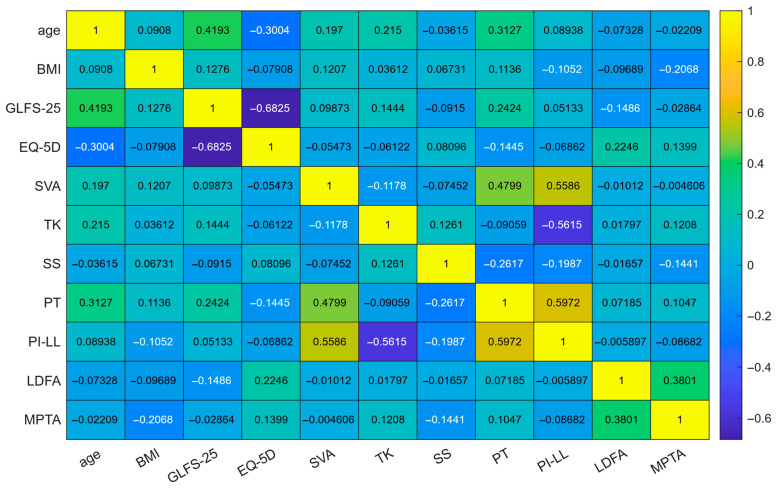
Spearman correlation matrix of baseline variables. Note: Colors encode Spearman’s correlation coefficients (yellow = positive, blue = negative). Numbers denote the coefficients. Abbreviations: BMI, body mass index; GLFS-25, 25-question Geriatric Locomotive Function Scale; EQ-5D, EuroQol 5-dimension; SVA, sagittal vertical axis; TK, thoracic kyphosis; SS, sacral slope; PT, pelvic tilt; PI–LL, pelvic incidence minus lumbar lordosis; LDFA, lateral distal femoral angle; MPTA, medial proximal tibial angle.

**Figure 3:**
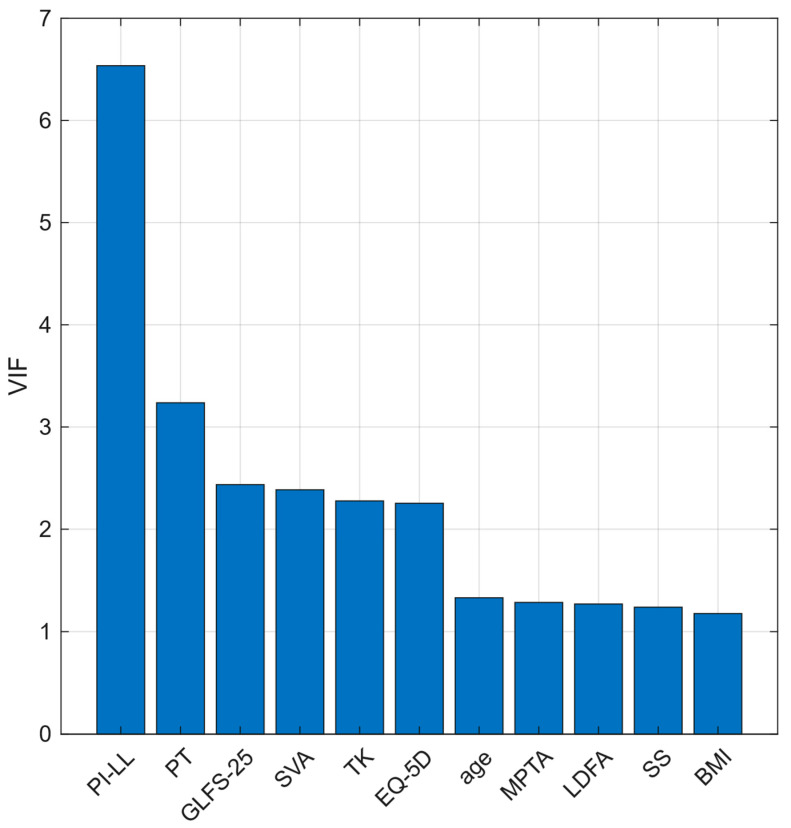
Variance inflation factors (VIFs) of baseline explanatory variables. Note: Higher VIF indicates stronger multicollinearity; PI–LL mismatch had the highest VIF. BMI had the lowest, indicating its contribution to independent information. Abbreviations: VIF, variance inflation factor; BMI, body mass index; GLFS-25, 25-question Geriatric Locomotive Function Scale; EQ-5D, EuroQol 5-dimension; SVA, sagittal vertical axis; TK, thoracic kyphosis; SS, sacral slope; PT, pelvic tilt; PI–LL, pelvic incidence minus lumbar lordosis; LDFA, lateral distal femoral angle; MPTA, medial proximal tibial angle.

BMI correlated modestly with MPTA and showed the lowest VIF. PI–LL mismatch correlated with multiple sagittal parameters (SVA, TK, SS, PT) and had the highest VIF (Figure 2 and Figure 3).

**Figure 4:**
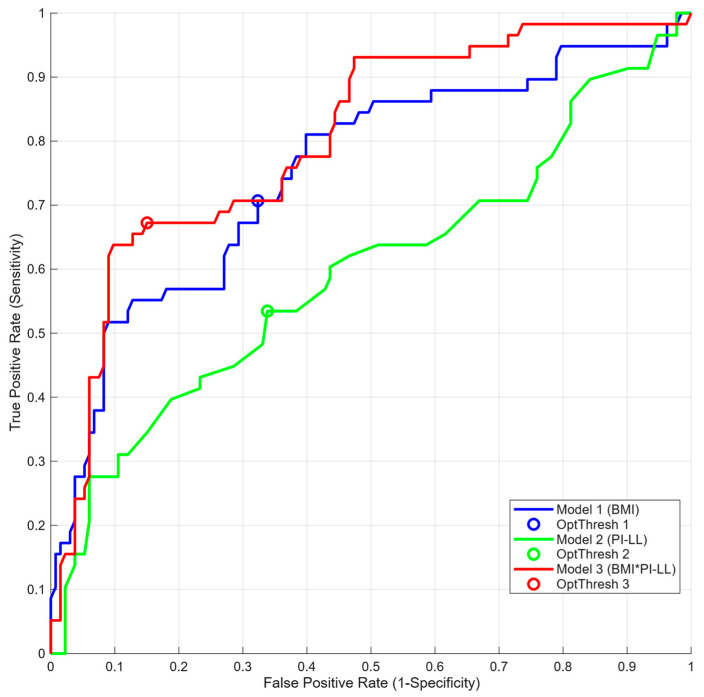
ROC curves for models predicting incident KOA. Note: The combined logistic model using body mass index (BMI) and pelvic incidence–lumbar lordosis (PI–LL) mismatch achieved the highest discrimination (AUC 0.803) compared with BMI alone (AUC 0.753) and PI–LL alone (AUC 0.596). The optimal probability cutoff was selected by the shortest Euclidean distance to the upper-left corner (0, 1). Abbreviations: ROC, receiver operating characteristic; AUC, area under the curve; BMI, body mass index; PI–LL, pelvic incidence minus lumbar lordosis; KOA, knee osteoarthritis.

In ROC analyses, the AUCs of BMI alone, PI–LL mismatch alone, and the combined model (BMI + PI–LL mismatch) were 0.753, 0.596, and 0.803, respectively (Table 3, Figure 4). The ROC-derived BMI cutoff was 22.07 kg/m^2^. The interaction model’s decision boundary was −0.4066 < −11.445 + 0.45543 × BMI + 0.28648 × PI–LL mismatch − 0.011004 × BMI × PI–LL mismatch.

**Figure 5:**
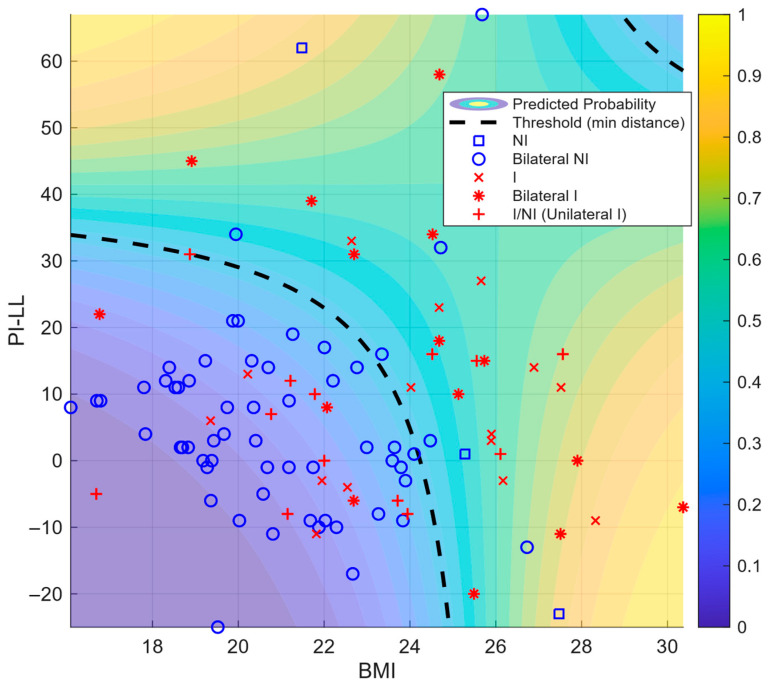
Scatter plot of BMI versus PI–LL mismatch overlaid with predicted probabilities. Note: Background heatmap represents model’s estimated probability of belonging to I group based on logistic regression including BMI, PI–LL, and their interaction term (**). Dashed line marks the probability cutoff minimizing Euclidean distance from ROC curve to point (0,1), representing optimal balance between sensitivity and specificity. Participants are colored red if either knee belonged to the I group. NI group = blue squares and circles; I group = red crosses and asterisks; squares and crosses indicate unilateral lesions; circles and asterisks indicate bilateral lesions. Red plus signs indicate overlapping I and NI observations sharing identical BMI and PI–LL coordinates in which overlapping points are plotted as unilateral I. ** (logit [P] = β_0_ + β_1_·BMI + β_2_·PI–LL + β_3_·BMI·PI–LL) Abbreviations: BMI, body mass index; PI–LL, pelvic incidence minus lumbar lordosis; NI, non-incident knee osteoarthritis group (KOA); I, incident KOA group; ROC, receiver operating characteristic.

A BMI–PI–LL mismatch heat map visually depicts the lower risk at jointly low values (Figure 5).

Within the multivariable framework, BMI remained the strongest single predictor of incident KOA. Adding a BMI × PI–LL mismatch interaction modestly improved discrimination over the BMI-only model (AUC 0.803 vs. 0.753; Table 3, Figure 4). The predicted-risk surface showed the lowest risk, where both BMI and PI–LL mismatch were low, and higher risk along two complementary bands—(i) higher PI–LL mismatch at lower BMI and (ii) higher BMI at lower PI–LL mismatch—consistent with a negative BMI × PI–LL mismatch interaction (β < 0). The upper-right region (high BMI and high PI–LL mismatch) was sparsely populated and did not exhibit the highest predicted risk (Figure 5). Thus, PI–LL mismatch operates as a complementary risk marker that improves risk stratification beyond BMI.

To address potential overfitting, we applied Harrell’s bootstrap optimism correction (1000 resamples) to the final interaction model. After correction, performance remained robust: AUC = 0.792; Brier score = 0.166; Brier Skill Score = 0.221; calibration slope = 0.925; calibration intercept = 4.586 × 10^−4^. These bootstrap-corrected metrics support the stability of the BMI × PI–LL interaction and reinforce its incremental value for risk stratification beyond BMI alone.

## 4. Discussion

This 8-year cohort study identified BMI as the strongest single predictor of incident KOA, with PI–LL mismatch contributing complementary value despite limited standalone discrimination. To reduce the confounding influence of two well-known risk factors—sex and prior joint injury [7,9]—only women were included, and participants with a history of lower limb surgery were excluded. Univariate differences (BMI, GLFS-25, EQ-5D, PT, PI–LL, MPTA, JLO) aligned with those of prior literature [6,7,10,17,24], whereas age was not a significant predictor, possibly reflecting KOA onset in midlife [25].

BMI is a well-established risk factor for KOA [6,7,10,24,26,27]. Even within a relatively lean cohort (mean BMI 21.9 kg/m^2^) [28], BMI retained strong predictive value, with a ROC-derived cutoff of 22.07 kg/m^2^. This heightened susceptibility to load-related stress may be attributed not only to the relatively advanced age of the participants but also to population-specific skeletal morphology and culturally specific lifestyle habits, such as frequent squatting and kneeling, which may predispose Japanese individuals to KOA compared with populations in other countries [17,29,30].

Prior studies have shown that greater PI–LL mismatch is observed in more advanced KOA and is linked to compensatory knee flexion [13,14,16,20,31]. In contrast, our cohort comprised knees with KL 0–1 at baseline and did not exhibit sagittal malalignment that was severe enough to necessitate compensation, indicating a different clinical context. Within this early-stage setting, PI–LL alone showed limited discrimination (AUC 0.596) and correlated with multiple sagittal parameters with the highest VIF, suggesting shared variance with global alignment. Thus, we interpreted these results to indicate that PI–LL is not a strong solo predictor or a direct interventional target. Rather, PI–LL serves as a complementary marker that improves risk stratification when considered in conjunction with BMI.

In keeping with this early-stage context, femoral inclination—a proxy for compensatory knee flexion—was not significantly associated with incident KOA in our data, implying that sagittal imbalance had not yet translated into measurable lower-limb compensation at baseline. Conceptually, sagittal spinopelvic malalignment may begin with a reduction in lumbar lordosis [32]; accordingly, monitoring PI–LL may facilitate early detection of emerging global sagittal imbalance and potential increases in knee joint loading.

Incorporating a BMI × PI–LL interaction improved discrimination versus the BMI-only model (AUC 0.803 vs. 0.753; Table 3, Figure 4). However, the risk surface did not peak where both BMI and PI–LL were high. Instead, risk increased along complementary patterns—higher PI–LL at lower BMI and higher BMI at lower PI–LL—consistent with a negative (antagonistic) interaction on the logit scale (β for BMI × PI–LL = −0.011004), whereby the effect of one predictor attenuates as the other increases.

Clinically, these results suggest that primary prevention should prioritize weight management [26]. Spinopelvic assessment (by PI–LL) can refine BMI-based screening, highlighting risk among lean individuals with sagittal malalignment and among individuals with higher BMI even when PI–LL is low. Trunk and lower-limb muscle weakness may contribute to PI–LL mismatch [33,34], and targeted exercise may improve spinopelvic alignment [34]. Whether modifying these features reduces incident KOA, however, warrants prospective evaluation.

Proximal tibial varus alignment has been implicated as an important biomechanical factor in KOA development [17,35]. In our cohort, the between-group difference in MPTA was <1°, which may reflect a selection effect whereby restricting enrollment to baseline early KOA (KL 0–1) probably underrepresented advanced cases with smaller MPTA, thereby attenuating the apparent contrast. The weak correlation of MPTA with BMI suggests that chronic load may contribute to varus remodeling. However, the clinical impact at baseline appeared small in our study population.

Although this study provides important evidence on predictors of incident KOA in older Japanese women, several limitations warrant mention. First, the two-wave design (2012 and 2020) prevents precise ascertainment of onset, so estimates may be conservative. Second, selection and attrition (e.g., exclusions due to image quality or incomplete questionnaires, and loss to follow-up) could introduce bias; detailed flow is provided, but residual selection bias cannot be excluded. Third, radiographic alignment measures (e.g., PI–LL) are subject to posture-related measurement error, which likely attenuates effects. Fourth, while BMI emerged as a dominant predictor, limited body-composition and muscle-function data constrained our ability to disentangle adiposity from sarcopenic components. Fifth, generalizability is limited to community-dwelling Japanese women aged ≥50 years in a rural setting; external validation is needed. Finally, although logistic regression does not assume normality of predictors, unmeasured or residual confounding (e.g., baseline knee symptoms, lifestyle and activity patterns, comorbidities) may still influence the estimates.

In summary, BMI primarily drives incident KOA risk, and PI–LL complements BMI to improve discrimination, supporting prevention strategies that prioritize weight while using spinopelvic metrics for risk refinement.

## 5. Conclusions

BMI was the primary predictor of incident KOA, even in a relatively lean population. PI–LL mismatch alone had limited discrimination but complemented BMI to improve prediction, consistent with sagittal-alignment-related mechanical stress. Prevention should prioritize weight management, using spinopelvic parameters to refine individual risk.

## Figures and Tables

**Table 1:** Baseline characteristics of the study population stratified by incident knee osteoarthritis (KOA) status (knee-level).

Variable	Overall	Non-Incident KOA	Incident KOA	*p*-Value
Age	69.3 ± 6.1	69.1 ± 6.0	69.5 ± 6.3	0.691
Height	150.1 ± 5.8	150.2 ± 5.9	149.6 ± 5.6	0.521
Body weight	49.4 ± 7.4	47.6 ± 5.9	53.4 ± 8.8	<0.001
BMI	21.9 ± 3.0	21.1 ± 2.5	23.8 ± 3.1	<0.001
One-leg standing time	46.0 ± 19.9	47.2 ± 19.3	43.3 ± 20.8	0.226
FRT	36.5 ± 6.5	36.4 ± 6.7	36.8 ± 5.9	0.694
GLFS-25	7.6 ± 9.6	6.2 ± 6.9	10.7 ± 13.6	0.022
EQ-5D	0.9 ± 0.1	0.9 ± 0.1	0.8 ± 0.2	0.02
SVA	31.6 ± 39.6	29.4 ± 37.9	36.7 ± 42.6	0.267
TK	34.8 ± 14.1	36.0 ± 13.9	31.9 ± 14.2	0.073
LL	42.5 ± 15.8	44.0 ± 14.5	39.0 ± 17.9	0.064
SS	30.6 ± 9.2	31.1 ± 8.2	29.3 ± 11.0	0.254
PT	18.8 ± 8.2	17.9 ± 8.1	20.8 ± 8.0	0.022
PI	49.7 ± 9.9	49.3 ± 10.0	50.5 ± 9.6	0.462
PI–LL	7.2 ± 16.0	5.3 ± 14.5	11.5 ± 18.3	0.026
FI	3.3 ± 4.4	2.9 ± 4.2	4.1 ± 4.6	0.08
LDFA	89.1 ± 2.6	89.3 ± 2.7	88.7 ± 2.3	0.131
MPTA	85.6 ± 2.1	85.8 ± 2.1	85.1 ± 2.1	0.032
aHKA	−3.5 ± 2.6	−3.5 ± 2.5	−3.6 ± 3.0	0.763
JLO	174.8 ± 3.9	175.1 ± 4.1	173.8 ± 3.3	0.022
HKA	2.6 ± 2.6	2.3 ± 2.2	3.1 ± 3.2	0.097
JLCA	−1.0 ± 1.8	−1.2 ± 1.8	−0.7 ± 1.8	0.083
FCI	−2.1 ± 2.8	−2.2 ± 3.0	−1.8 ± 2.5	0.311
TPI	1.1 ± 2.6	1.1 ± 2.7	1.1 ± 2.3	0.887

Note: Values are presented as mean ± standard deviation. *p*-values were calculated using Student’s *t*-test for continuous variables. Abbreviations: BMI, body mass index; FRT, functional reach test; GLFS-25, 25-question Geriatric Locomotive Function Scale; EQ-5D, EuroQol 5-dimension; SVA, sagittal vertical axis; TK, thoracic kyphosis; LL, lumbar lordosis; SS, sacral slope; PT, pelvic tilt; PI, pelvic incidence; PI–LL, pelvic incidence minus lumbar lordosis; FI, femoral inclination; LDFA, lateral distal femoral angle; MPTA, medial proximal tibial angle; aHKA, arithmetic hip–knee–ankle angle; JLO, joint line obliquity; HKA, hip–knee–ankle angle; JLCA, joint line convergence angle, FCI, femoral condyle inclination; TPI, tibial plateau inclination.

**Table 2:** Multivariable logistic regression analysis incorporating a BMI × PI–LL interaction.

Variable	β	95% CI (β) Lower	95% CI (β) Upper	OR	95% CI (OR) Lower	95% CI (OR) Upper	*p*-Value
(Intercept)	−11.445	−15.293	−7.597	0.00001	2.283	0.0005	<0.001
BMI	0.455	0.291	0.619	1.577	1.338	1.858	<0.001
PI–LL	0.286	0.087	0.486	1.332	1.091	1.625	0.005
BMI × PI–LL	−0.011	−0.019	−0.003	0.989	0.981	0.997	0.010

Note: The logistic value was calculated using the equation: −11.445+0.45543×BMI+0.28648×PI–LL−0.011004×BMI×PI–LL. Abbreviation: BMI, body mass index; PI–LL, pelvic incidence minus lumbar lordosis; β, regression coefficient; CI, confidence interval; OR, odds ratio.

**Table 3:** Performance of uni- and multivariable logistic-regression models predicting incident KOA using BMI, PI–LL mismatch, and their interaction.

Logistic Model (Variables)	AUC	Cut-Off Probability	Cut-Point Value	TPR	FPR
β_0_ + β_1_·BMI	0.7529	0.2791	22.0667	0.7069	0.3233
β_0_ + β_1_·PI–LL	0.5956	0.3126	10.0000	0.5345	0.3383
β_0_ + β_1_·BMI + β_2_·PI–LL + β_3_·BMI·PI–LL	0.8027	0.3997	N/A	0.6724	0.1504

Note: This table reports the area under the receiver operating characteristic [ROC] curve (AUC), the probability threshold (cut-off) that minimizes the Euclidean distance from the ROC curve to the point (0,1), the corresponding cut-off value on the predictor scale, and the true positive rate (TPR: sensitivity) and false positive rate (FPR: 1—specificity) at that threshold. Here, each β coefficient represents the change in the log-odds of the outcome associated with a one-unit increase in the corresponding predictor, with β_0_ denoting the model intercept. In-text values are rounded to two decimals; tabled values to four decimals; all computations used full precision. Abbreviations: β, regression coefficient; BMI, body mass index; PI–LL, pelvic incidence minus lumbar lordosis; AUC, area under the curve; ROC, receiver operating characteristic.

## Data Availability

The dataset contains potentially identifying information from a small community-based cohort and is therefore not publicly available. De-identified data and the MATLAB analysis scripts used in this study are available from the corresponding author on reasonable request and with permission of the Institutional Review Board of Hamamatsu University School of Medicine (approval code 25-061, approval date 7 July 2025). Raw radiographic images cannot be shared publicly due to privacy and ethical restrictions.

## Abbreviations

The following abbreviations are used in this manuscript:

BMI	Body mass index
FRT	Functional reach test
GLFS	Geriatric Locomotive Function Scale
EQ-5D	EuroQol 5-dimension
SVA	Sagittal vertical axis
TK	Thoracic kyphosis
LL	Lumbar lordosis
SS	Sacral slope
PT	Pelvic tilt
PI	Pelvic incidence
PI–LL	Pelvic incidence minus lumbar lordosis
FI	Femoral inclination
LDFA	Lateral distal femoral angle
MPTA	Medial proximal tibial angle
aHKA	Arithmetic hip–knee–ankle angle
JLO	Joint line obliquity
HKA	Hip–knee–ankle angle
JLCA	Joint line convergence angle
FCI	Femoral condyle inclination
TPI	Tibial plateau inclination
Β	Regression coefficient
CI	Confidence interval
OR	Odds ratio
AUC	Area under the curve
VIF	Variance inflation factor
KOA	Knee osteoarthritis
ROC	Receiver operating characteristics
